# Novel Carbonaceous Adsorbents Prepared from Glycerin Waste and Dopamine for Gas Separation

**DOI:** 10.3390/molecules28104071

**Published:** 2023-05-13

**Authors:** Mary Batista, Renato Carvalho, Moisés L. Pinto, João Pires

**Affiliations:** 1 CQE, Centro de Química Estrutural, Institute of Molecular Sciences, Departamento de Química e Bioquímica, Faculdade de Ciências, Universidade de Lisboa, Campo Grande, 1749-016 Lisboa, Portugal; mkbatista@fc.ul.pt; 2 IBEROL, Sociedade Ibérica de Biocombustíveis e Oleaginosas, S.A., 2600-531 Alhandra, Portugal; renato.carvalho@iberol.pt; 3 CERENA, Departamento de Engenharia Química, Instituto Superior Técnico, Universidade de Lisboa, 1049-001 Lisboa, Portugal; moises.pinto@tecnico.ulisboa.pt

**Keywords:** glycerin, dopamine, activated carbon, ethane, ethylene, adsorption

## Abstract

Glycerin, a low-valued waste from biodiesel production, and dopamine were used as precursors for adsorbent materials. The study is centered on the preparation and application of microporous activated carbon as adsorbent materials in the separation of ethane/ethylene and of gases that are natural gas or landfill gas components (ethane/methane and carbon dioxide/methane). The activated carbons were produced by the following sequence reactions: facile carbonization of a glycerin/dopamine mixture and chemical activation. Dopamine allowed the introduction of nitrogenated groups that improved the selectivity of the separations. The activating agent was KOH, but its mass ratio was kept lower than one to improve the sustainability of the final materials. The solids were characterized by N_2_ adsorption/desorption isotherms, SEM, FTIR spectroscopy, elemental analysis, and point of zero charges (pH_PZC_). The order for adsorption of the different adsorbates (in mmolg^−1^) on the most well performing material—Gdop0.75—is methane (2.5) < carbon dioxide (5.0) < ethylene (8.6) < ethane (8.9).

## 1. Introduction

The last years have seen significant progress in the field of purification and separation of gaseous mixtures due to the development and use of nanoporous adsorbents such as activated carbons. These are materials with a well-developed porous structure, extensively used for the adsorption of several environmental contaminants, catalysis, and gas separation [1,2,3,4,5]. The broad application of activated carbons is due to their tunable textural properties such as surface area, pore volume, and pore size distribution. Low-cost activated carbons may be produced from residues that have poor economic value but can obtain adsorbents with properties similar to, or even better than, those of the activated carbons currently available in the market. In general, abundant and inexpensive materials with high carbon and low inorganic content are suitable as activated carbon precursors [1]. In the past years, a very large array of nanoporous adsorbents was explored for the separation of gases. The ethane/ethylene separation, for example, is one of the most energy-intensive single distillations performed commercially [6,7]. To reduce the ethylene production cost, it is highly desired to develop new separation methods—for example, the adsorption separation. However, most adsorbents show preferential adsorption to the alkene molecule which makes the ethane/ethylene separation by adsorption not cost effective, as discussed elsewhere [7,8]. In this sense, it is imperative to develop alkane-selective adsorbents with high alkane uptake and high selectivity values [6,7]. Some ethane-selective materials, such as some Metal–Organic Frameworks (MOFs), for instance [8,9], were described in the literature. Nevertheless, issues related to the cost and environmental sustainability of the production of adsorbents to be used in such a large-scale process as the ethane/ethylene separation are not entirely clear, and give space to the development of low-cost adsorbents produced from wastes as activated carbons. Other relevant separations that can potentially be made by adsorption using large amounts of adsorbent material are the methane/ethane separation connected with the recovery of ethane from natural gas, particularly when related with compressed natural gas [10] and, also, the carbon dioxide/methane separation in biogas, mostly landfill gas, so that methane can be used as fuel (gas upgrading). In fact, the amount of methane in landfill gases ranges from 45 to 60% and carbon dioxide from 40 to 55% [11]. The recovery of methane from landfill gas is particularly important. If not recovered, methane is released from the landfills into the atmosphere, and has an equivalent greenhouse effect that is more than 80 times higher than carbon dioxide. Furthermore, evidence for the prudent use and more local production of methane globally has been reported recently.

Much effort has been made to synthesize and tailor the microstructures of porous carbon materials via various activation procedures (chemical or physical activation). For instance, the chemical activation of various carbon sources using KOH as the activating reagent is promising because of its lower activation temperature and high yields, well-defined micropore size distribution, and high specific surface area of the resulting porous carbons [12,13]. Additionally, the enrichment of the activated carbons in nitrogen functional groups is of particular interest for the improvement of the separation selectivity of the gas systems mentioned above. In fact, polydopamine and glucosamine carbon materials were reported as adsorbent materials for gas separation, inclusively promoting the ethane selectivity [14,15].

To our knowledge, this is the first study on the preparation, characterization, and performance of activated carbons prepared from an industrial waste (in this case, glycerin) modified with dopamine. Dopamine was used here as the source of nitrogen functional groups. Glycerin waste is both an economical and an environmental issue [16]. The biodiesel industry is pushed to increase production originating wastes and residues that have several contaminants to be considered as raw materials and, because of this, many applications of glycerin tend to be avoided due to a lack of guarantees in the purification processes to remove or even destroy those contaminations. Applications such as food or feed, pharmaceutical, and cosmetics are normally rejected, and simple technical/chemical ones are considered. The efficient conversion of glycerin—of which waste amounts to 10% of the produced biodiesel and so its accumulation is becoming an environmental concern— into valued products would contribute positively towards the biodiesel economy [17]. In the present work, the prepared glycerin–dopamine activated carbon adsorbents prepared were evaluated for the separation of gas mixtures (ethane/ethylene, ethane/methane, or carbon dioxide/methane).

## 2. Results and Discussion

### 2.1. Characterization of the Adsorbents

**Figure 1:**
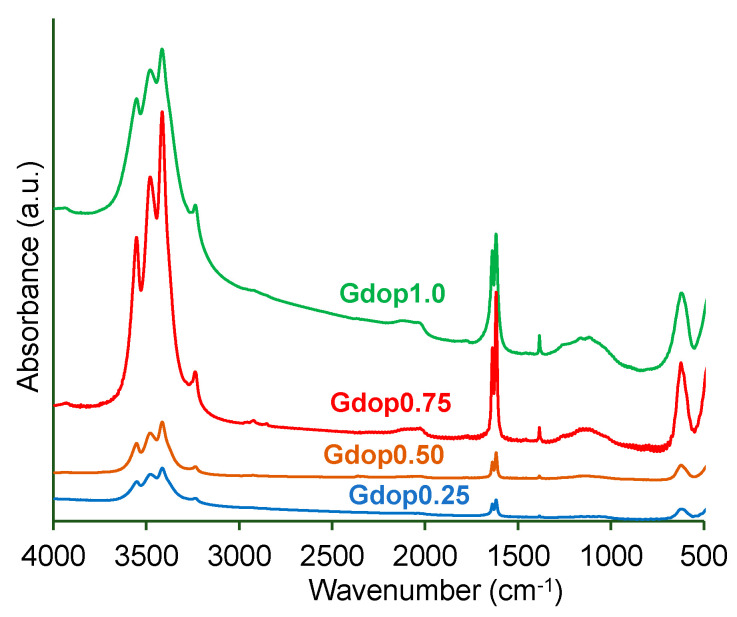
FTIR spectra for the various activated carbons.

In Figure S3a in the Supporting Information, the glycerin–dopamine carbonized (GdopC) FTIR-spectrum presents functional peaks at 3544 cm^−1^ (N-H stretching vibration); 3461 cm^−1^ (O-H stretching); 3408 cm^−1^ (aromatic C-H stretching vibration); aliphatic asymmetric and symmetric C-H stretching vibrations at 2952 cm^−1^, 2933 cm^−1^, and 2834 cm^−1^, respectively; and a peak in 1621 cm^−1^ that is indicative of the presence of N-H bending vibration and 1616 cm^−1^ (aromatic C-C stretching vibration) [18,19]. The peak at 1384 cm^−1^ corresponds to the stretching vibration of -SO_3_ [18,19] which is expected since sulfuric acid was used in the preparation of the glycerin–dopamine carbonized. The SEM-image in Figure S3b shows that GdopC presents a compact morphology formed of spherical and irregular shapes, consistent with dopamine and glycerin–carbon materials, respectively, according to the literature [4,20]. Furthermore, the obtained N_2_ adsorption isotherm for GdopC (not presented) corresponds to an isotherm characteristic of a non-porous material with *A*_BET_ ≈ 10 m^2^ g^−1^ in line with previous results [2,4]. All FTIR-spectra for the glycerin–dopamine-activated carbons—Figure 1—exhibit similar peaks. The broad absorption bands at around 3560 cm^−1^ can be attributed to the N-H stretching vibration of the O-H stretching vibration, and the band at 3406 cm^−1^ represents the aromatic C-H stretching vibration. The existence of the sulfuric group (-SO_3_H) in the glycerin derivatives was further confirmed by the presence of a broad absorption band at 3461 cm^−1^ which corresponds to the S-H stretching vibration [18,19]. The weak bands at 2975 cm^−1^, 2935 cm^−1^, and 2859 cm^−1^ are attributed to symmetric and asymmetric aliphatic C-H stretching vibrations. Moreover, stretching vibrations were also observed around at 1384 cm^−1^ [18,19]. The presence of a band at 1384 cm^−1^ is indicative of S-O stretching vibrations (due to the presence of sulfate or sulfonyl groups) which is anticipated since sulfuric acid was used in the preparation of the carbonized [18]. These results can be supported by the data obtained by elemental analysis.

The results of the elemental analysis in Table 1, in conjunction with the infrared spectroscopy data discussed above, were used to characterize the surface chemistry of the materials prior to and after the activation processes. The chemical composition of the glycerin in Table 1 presents a high carbon content (∼39%), although—and as expected—this is lower than the values found for solid wastes [21], making it a suitable precursor for obtaining activated carbons. The activated carbons obtained have a high carbon content (62–75%) compared with the carbon content for other activated carbons prepared from various types of waste as reviewed recently [22].

The results given in Table 1 suggest that the acidic character of the activated carbons, as indicated by the pHpzc values, is mainly due to the incorporation of oxygen-containing surface groups during the activation with potassium hydroxide. 

**Figure 2:**
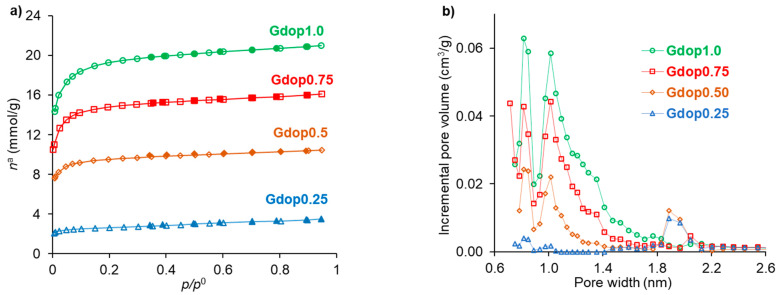
(**a**) N_2_ adsorption–desorption isotherms (desorption points are represented by closed symbols); (**b**) Micropore-size distributions obtained from the DFT method.

Nitrogen adsorption–desorption isotherms for all activated carbons are shown in Figure 2a, which can be identified as type I according to the IUPAC classification [23] and are characteristic of materials with developed microporosity. Table 2 summarizes the textural properties for all activated carbons which have apparent surface area values up to 1657 m^2^ g^−1^ and total pore volume values in the range of 0.12–0.73 cm^3^ g^−1^. The presence of micropores in the activated carbons can be clearly verified by their pore-size distribution in Figure 2b. The character bi-modal of the distributions in Figure 2b is a feature common to various types of activated carbons [24].

The yield and apparent-tap-density were affected by the KOH:GdopC ratios (Table 1). Higher amounts of KOH consistently gave products with larger apparent surface area (*A*_BET_) as well as pore volumes, but with lower apparent-tap-densities, particularly for the sample Gdop1.0. Interestingly, when comparing the samples in Table 1 with the materials activated at the same temperature and with the same ratio, KOH:char, but without using dopamine [4], these samples (without dopamine) presented smaller values of BET surface area and microporous volume than the sample prepared in the present work with dopamine.

**Figure 3:**
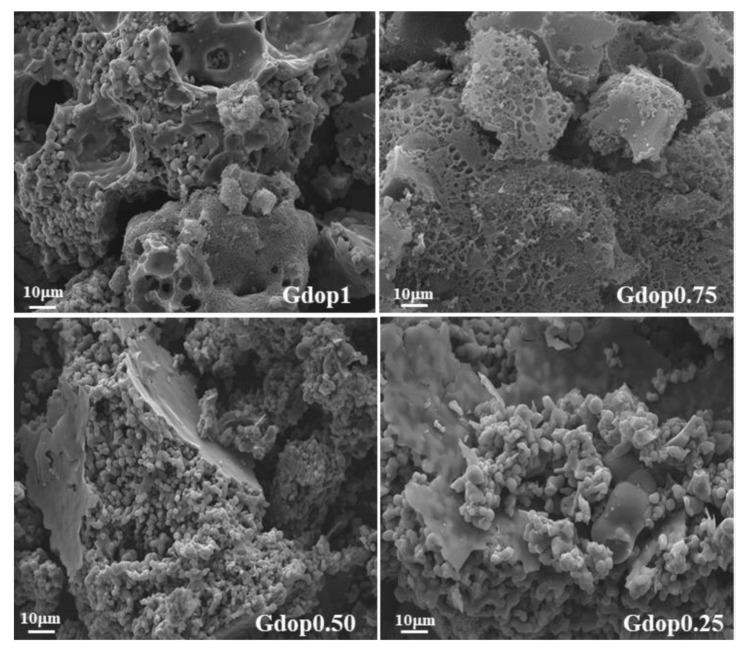
SEM micrographs for the indicated activated carbons.

The morphology of the activated carbons was investigated by SEM, shown in Figure 3. The glycerin–dopamine carbonized (Figure S3b) shows a powder with compact morphology whereas, after the activation process, in the cases of Gdop1.0 and Gdop0.75, these materials adopt a more sponge-like aspect, with some heterogeneity in the particle sizes. In the cases of Gdop0.50 and Gdop0.25, the morphology is more similar to GdopC. These observations are in line with the apparent-tap-density values that are higher for the materials that were activated with the lower proportions of KOH.

### 2.2. Adsorption of Ethane, Ethylene, Carbon Dioxide, and Methane

**Figure 4:**
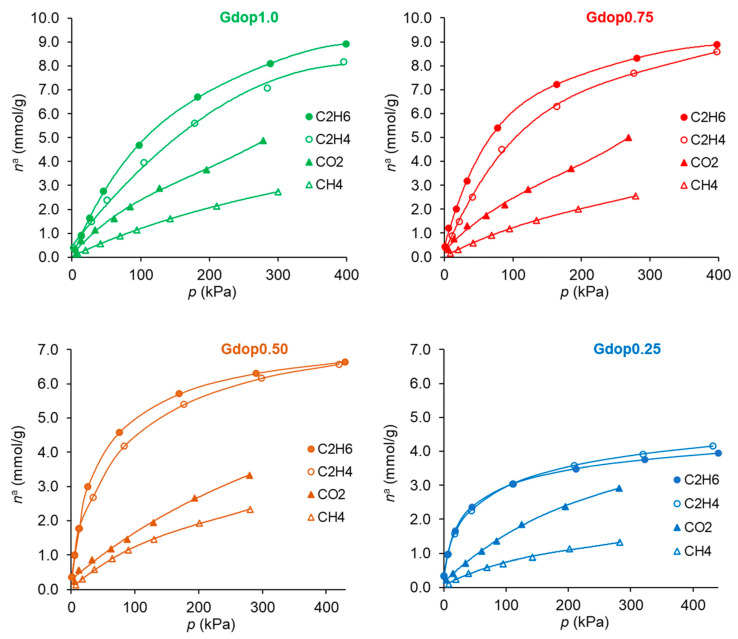
Adsorption isotherms of the studied gases at 25 °C on the activated carbons.

The adsorption isotherms of ethane, ethylene, carbon dioxide, and methane at 25 °C in the prepared glycerin-activated carbons (Gdop1.0, Gdop0.75, Gdop0.5, and Gdop0.25) are shown in Figure 4 for pressures up to 400 kPa. In the case of the ethane/ethylene, it can be seen from Figure 4 that, except for the material prepared with the lowest amount of activating agent—that is, the sample Gdop0.25—all activated carbons adsorb higher amounts of ethane than ethylene or, in other words, the materials are ethane-selective adsorbents. From the technological point of view, this is of major importance since, with ethane-selective adsorbents, the most valuable molecule (ethylene) is obtained first from the adsorption cycle [25,26]. A comparison of the amounts of ethane and ethylene adsorbed by the materials in the present work with results from the literature can be drawn. This comparison makes sense only for other ethane-selective materials, mostly activated carbons and some MOFs. Selecting values at atmospheric pressure for comparison, the literature values range between 0.9 and 7.9 mmolg^−1^, as recently reviewed [6,26], being the average value near 4 mmolg^−1^ (modal value 3.3 mmolg^−1^) which is less than the amount of ethane adsorbed in the best material of the present study (Gdop0.75): 5.4 mmolg^−1^. The ethane selectivity in the active carbons is expected to result mostly from interactions by dispersion forces. In fact, not only does ethane have a high polarizability [27] but it also has more possibilities to promote interactions between the H atoms, in particular with the N and O atoms at the surface. These interactions will occur between the electropositive C-H bonds and the electronegative N and O at the surface [14]. In this way, ethane may potentially form more C-H∙∙∙O or C-H∙∙∙N interactions with the glycerin–dopamine activated carbons since it contains six electropositive C-H bonds, whereas ethylene only contains four electropositive C-H bonds, resulting in the stronger interaction of ethane with the surfaces of the glycerin–dopamine activated carbons compared to ethylene.

**Figure 5:**
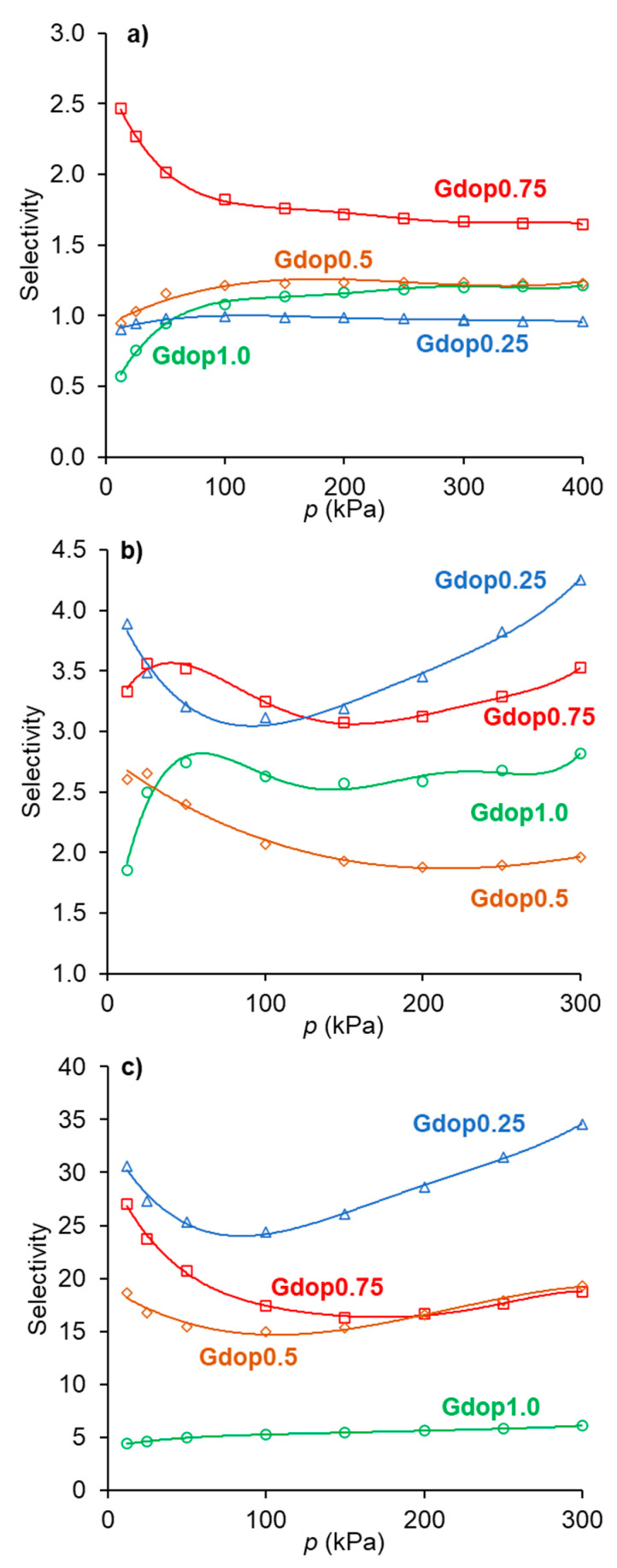
Selectivity values obtained from the IAST calculations for the separations (**a**) C_2_H_6_/C_2_H_4_; (**b**) CO_2_/CH_4_; and (**c**) C_2_H_6_/CH_4_ in the various activated carbons.

**Figure 6:**
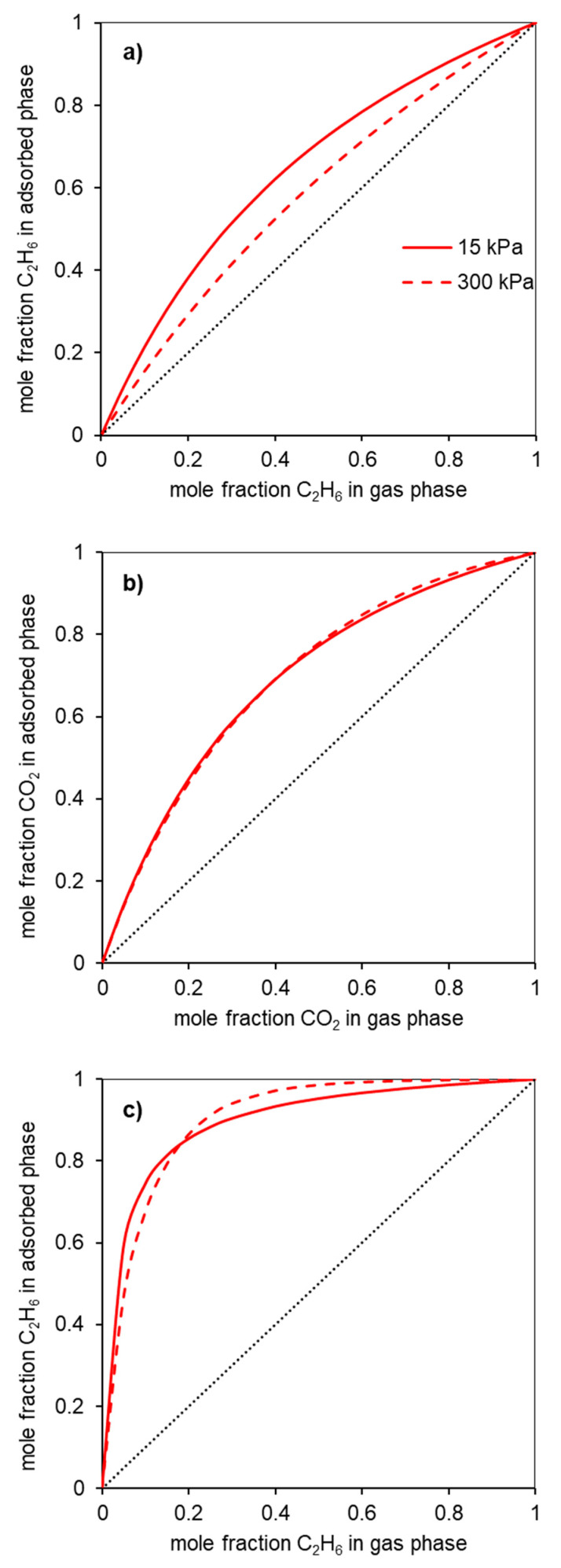
X-Y diagrams estimated from the Ideal Adsorbed Solution Theory for the separations (**a**) C_2_H_6_/C_2_H_4_; (**b**) CO_2_/CH_4_; and (**c**) C_2_H_6_/CH_4_ in the sample Gdop0.75. The data are exemplified at two pressures: 15 (solid line) and 300 kPa (dashed line). The dotted line would imply no separation (separation selectivity = 1).

The selectivity values are an important magnitude to evaluate the potentialities of a given adsorbent for a particular separation. Various methodologies can be used to obtain the selectivity values (or separation factors). One of the methodologies most used for this is the Ideal Adsorbed Solution Theory (IAST) which was developed by Myers and Prausnitz [28]. This theory is based on the solution thermodynamics and is independent of an actual model of adsorption [29]. A Python package of software was developed to perform IAST calculations and predict mixed-gas adsorption isotherms from the pure component adsorption isotherms [30]. The selectivity values in the function of the pressure in Figure 5, and the gas phase/adsorbed phase composition diagrams (X-Y diagrams) for a given pressure in Figure 6, were obtained in this way.

For the ethane/ethylene separation, the selectivity values (Figure 5a) are higher than one (ethane-selective materials) for all samples except for the material activated with the lower amount of activating agent (Gdop0.25). This was already anticipated from the isotherms in Figure 4. The most favorable selectivity is presented by the material, Gdop0.75, for which the selectivity decreases with the pressure and ranges from 2.5 to 1.7. Interestingly, a recent work based on computer simulation pointed out that, for materials in which the interactions of ethane and ethylene are mainly through dispersion forces, the maximum selectivity value that can be achieved is near 2.8 [26]; that is, close to the maximum values obtained with the Gdop0.75 material. According to Figure 5a, the increase in the activating agent proportion improves the ethane/ethylene selectivity, since the amount of Oxygen and Nitrogen groups also increase and, hence, the interactions with ethane are promoted. Nevertheless, this is not the case of the sample obtained with the highest KOH proportion—Gdop1.0. In fact, this material presents the highest specific surface area and microporous volume of the series at the cost of a stronger activation and the development of a higher proportion of the narrowest micropores. Very narrow micropores may not be beneficial for the ethane selectivity since ethane has a larger critical diameter than ethylene—4.44 and 4.16 Å, respectively [27]. Additionally, the Gdop1.0 sample presents the lowest value of apparent-tap-density, more than three times lower than the value for Gdop0.75, meaning that Gdop1.0 is a very light powder which, for adsorption applications, is not usually preferred.

Figure 6a presents the gas phase/adsorbed phase composition diagrams for the material Gdopo0.75-, for pressures below and above the atmospheric pressure, 15 and 300 kPa, respectively. It can be seen from this diagram that the material Gdop0.75 is ethane-selective in all the ranges of gas phase compositions at the indicated pressures. 

In the case of the CO_2_/CH_4_ systems, the adsorption isotherms (Figure 4) present the usual trend for most materials; that is, the amounts adsorbed of CO_2_ are higher than those of CH_4_. This is a consequence not only of the higher polarizability of CO_2_ than of CH_4_ (29.1 and 25.4 × 10^−25^ cm^3^, respectively [27]) but also due to the specific interactions that can be developed with the CO_2_ quadrupole moment (4.3 × 10^−26^ esu cm^2^, [27]). The latter interactions were pointed out to be favored in the case of nitrogen-rich activated carbons. The CO_2_-adsorbed amounts for the activated carbons prepared in the present work, at the maximum pressure studied (300 kPa), range from 2.9 mmolg^−1^ for the Gdop0.25 material to 5 mmolg^−1^ in the Gdop1.0 and Gdop0.75 samples. The selectivity values for Figure 5b have an apparent anomaly in their sequence since the less activated material—Gdop0.25— presents high selectivity values at a considerable range of pressures. Nevertheless, this situation is only apparent since, compared with the other materials, Gdop0.25 presents the least amounts adsorbed, and the differences between the CO_2_ and CH_4_ isotherms occur at the expense of a proportional larger decrease in the amounts adsorbed of CH_4_ in relation to the decrease of amounts adsorbed of CO_2_. In fact, because the Gdop0.25 has the lowest amount of Oxygen groups, the interactions via dispersion forces are reduced, but CO_2_ can additionally have interactions with the quadrupole, which are absent for methane. Concerning the remaining activated carbons, the most favorable material is the Gdop0.75, as was the case for the ethane/ethylene system, as a result of its surface chemistry and porosity. The X-Y diagram in Figure 6b for Gdop0.75 shows that this material is CO_2_-selective in all ranges of gas phase composition and, also, that it is not very dependent on the total pressure in the studied range of 15 to 300 kPa, since both curves are almost coincident. The comparison of the amounts adsorbed and the selectivity values for Gdop0.75 (Figure 4 and Figure 5b) with the literature values can be undertaken by considering the large amount of data for the adsorption of CO_2_ and CH_4_ published in recent reviews [31,32,33,34]. A wide range of values can be found in these works even if restricting the comparison to results obtained for experimental conditions and methodologies that are relatively similar to those used in our study. In this way, the amounts of CO_2_ adsorbed can vary from 1.9 to 7.9 mmolg^−1^, and those for CH_4_ from 0.4 to 2.5 mmolg^−1^ [31,32,33,34]. Concerning selectivity, the range of values in the literature is also considerable so it would perhaps be more instructive to compare them with the results from commercial-activated carbons. In this situation, selectivity values between 1 and 4.5 were reported [34].

For the third type of separation analyzed, the C_2_H_6_/CH_4_ system, the selectivity values ae presented in Figure 5c. The high selectivity values of Gdop0.25 occur due to the reasons previously discussed and, hence, the most favorable adsorbent is the Gdop0.75 sample, as was also the case for the C_2_H_6_/C_2_H_4_ and CO_2_/CH_4_ systems. The C_2_H_6_/CH_4_ separation is less studied in the literature. Nevertheless, the published results in carbon materials presented both lowest amounts adsorbed and lowest selectivity values than those obtained in the present work for the Gdop0.75 material [35].

A process still related with the gas molecules studied in the present work is the oxidative coupling of methane to produce ethylene [7,36]. The principal component from which ethylene needs to be separated in this process is carbon dioxide. The estimated selectivity values (between 2.4 and 3) and the X-Y diagrams are given in the Supporting Information, Section S3.

**Figure 7:**
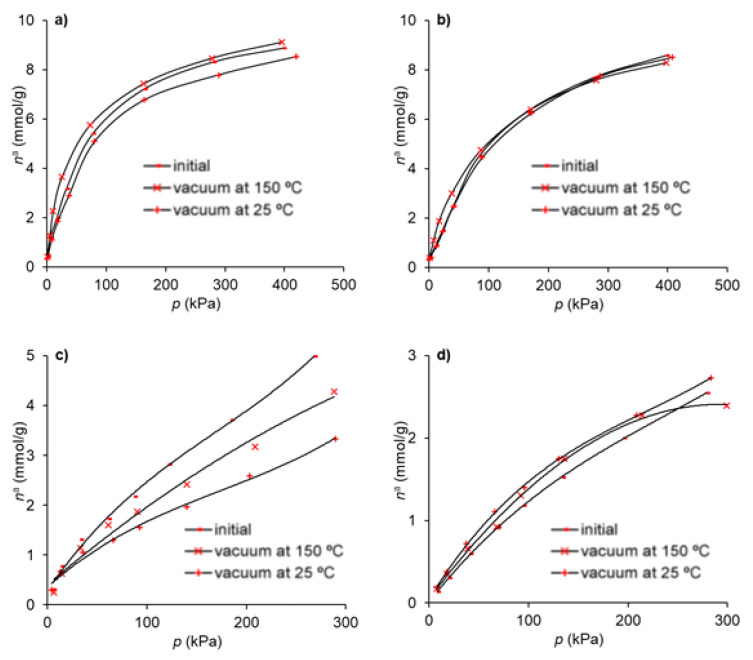
Adsorption isotherms in Gdop0.75: (**a**) ethane (**b**) ethylene (**c**) CO_2_ and (**d**) CH_4_ after regeneration under vacuum at 25 °C and 150 °C, compared with the initial results.

The regeneration of the adsorbents was carried out to investigate the effect on the adsorption capacity after a previous adsorption run. In this way, the effect of two consecutive adsorption cycles was assessed on the material that presents the best adsorption and selectivity characteristics as discussed above—that is, the Gdop0.75 material. The regeneration was achieved under vacuum, and two temperatures were tested—25 and 150 °C. The results are presented in Figure 7.

It can be seen from Figure 7 that, in the case of CO_2_, the temperature has a strong effect on the regeneration of the adsorbent capacity and that, for the highest temperature, the loss in the capacity is near 14%. For the remaining gases, the regeneration under vacuum does not depend significantly on the temperature used and the reduction in the adsorption capacity is lower than 7%.

## 3. Materials and Methods

### 3.1. Preparation of the Adsorbents

#### 3.1.1. Overview

The glycerin–dopamine activated carbons were prepared by the activation of glycerin–dopamine carbonized (GdopC) materials. The experimental methods of preparation of the carbonized and also of the activated carbons are described in the Supporting Information, Section S1. According to the results of optimization carried out in previous works, the activation temperature selected was 700 °C [4]. The samples were identified by the KOH:GdopC ratio. For instance, the label for the activated carbon Gdop1.0 means that a given amount of carbonized GdopC was activated with a mass ratio of KOH:GdopC ratio of 1. To improve the sustainability of the final material—namely, by reducing the amount of activating agent— the samples were prepared with a KOH:GdopC ratio equal or less than 1.0. In this way the materials Gdop1.0, Gdop0.75, Gdop0.5, and Gdop0.25 were prepared having the KOH:GdopC ratios of 1.0, 0.75, 0.50, and 0.25, respectively.

#### 3.1.2. Preparation of the Glycerin–Dopamine Carbonized

The GdopC was prepared by hydrothermal synthesis using a mixture of crude glycerin 82% glycerin supplied by a Portuguese company, dopamine 99% (ACROS Organics), and sulfuric acid 96% (Sigma-Aldrich, St. Louis, MO, USA). Briefly, a mixture of crude glycerin (10 g) and dopamine (1 g) was stirred at room temperature for 24 h. Then, sulfuric acid (a volume ratio glycerin:sulfuric acid of 1:0.5) was added, and the mixture was stirred at room temperature for ~30 min (until foaming ceased). Then, the mixture was transferred to a Teflon-lined stainless-steel autoclave and the acid carbonization was carried out at 180 °C for 6 h in an oven, Medline Scientific Limited, model ON-02G. The GdopC was washed with distilled water (water until the washing was neutral) and dried at 100 °C.

#### 3.1.3. Chemical Activation 

The activated carbons were obtained by chemical activation of glycerin–dopamine carbonized, with potassium hydroxide 99% (Aldrich). The GdopC was soaked with the adequate potassium hydroxide amount dissolved in 10 cm^3^ of distilled water, followed by stirring for 2 h at room temperature, and then dried at 100 °C. The temperature of activation was 700 °C and KOH:GdopC weight ratios (between 0.25:1 and 1:1). The mixture was activated in a horizontal furnace (Thermolyne, model 21100) under N_2_ flow (5 cm^3^ s^−1^) with the temperature raised (10 °C∙min^−1^) up to the activation temperature and kept for 1 h. After the chemical activation process, post-chemical activation was required to remove excess activating agent from the activated carbons. The glycerin-activated carbons were treated with HCl solution (1M), then washed with hot distilled water until the washing was neutral, and dried at 100 °C to allow the evaporation of the excess water. 

A schematic illustration of the preparation of the glycerin–dopamine carbon materials is presented in the Supporting Information, Section S1.

Calculation of yields: (i) carbonized Yield = [ (mass of char)/(mass of glycerin + dopamine)] × 100; (ii) activation Yield = [mass of activated carbon/(mass of char), and (iii) global Yield = [mass of activated carbon/(mass of glycerin + dopamine)] × 100. 

Apparent-tap-density (ratio of mass-to-tap volume) of all carbons was recorded according to a methodology adapted from the literature [4]. Briefly, a graduated cylinder of 5.0 mL and an accuracy of ± 0.1 mL was filled with 0.5 g (Mettler Toledo AB204-S/Fact) of the powdered activated carbon. Then, the cylinder was manually vibrated until the volume of the activated carbon remained constant for 2 min (tap volume).

### 3.2. Characterization of the Adsorbents

The surface functional groups of the adsorbent materials were determined by FTIR, Elemental analysis, and pH_PZC_. The FTIR analyses were performed by mixing dried carbons with KBr in a 1:100 weight ratio which was then ground into fine powder using a Nicolet 6700 FTIR spectrometer. The spectra were obtained in the wavenumber range between 4000 and 400 cm^−1^ (64 scanning; 4 cm^−1^ of resolution). The elemental analysis (CHNOS) of the GDMs was carried out in a CHNS Analyzer—Thermo Finnigan Flash, EA, 1112 series. The oxygen content was obtained by the difference between the total percentage (100%) and the sum of the percentages (wt%) of carbon, hydrogen, sulfur, and nitrogen. The pH measurements were taken with a Metrohm 744 pH Meter at the point of zero charges (pH_PZC_), adapting a methodology previously published [37]. 

Nitrogen adsorption isotherms at low temperature (−196 °C) were used for the textural characterization of the adsorbent materials. Nitrogen data were obtained in automatic equipment (NOVA 2200e, Quantachrome). Before the measurements, the samples (about 50 mg) were heated and outgassed (under vacuum better than 10^−2^ Pa) at 120 °C overnight. Apparent surface areas (*A*_BET_) were calculated using the N_2_ adsorption isotherm data within the relative pressure of 0.05–0.15 [25,38]. The *A*_BET_ values so obtained agreed within 1% with those obtained by a methodology recently proposed adopting more elaborated criteria. The total pore volume was determined according to the N_2_ volume at a relative pressure (*p*/*p*^0^) around 0.95 [19]. The micropore volume was calculated by the αs method, taking as reference the isotherm reported in the literature [39]. 

The morphology of the samples was observed by Scanning Electron Microscopy (SEM) performed on a Zeiss Supra 55 VP apparatus using 5 kV as the accelerating voltage. The apparent tap density of all materials was recorded as the ratio of mass-to-tap volume according to the methodology described previously [4].

### 3.3. Adsorption of Ethane, Ethylene, Carbon Dioxide, and Methane

The adsorption isotherms of C_2_H_6_ (Air Liquide, 99.995%), C_2_H_4_ (Matheson, 99.995%), and CO_2_ (Air Liquide, 99.99%) were measured at 25 °C in a custom-made volumetric apparatus of stainless steel, which comprises a pressure transducer (Pfeiffer Vacuum, APR 266) and a vacuum system that allows a vacuum better than 10^−2^ Pa (schematic diagram in the Supporting Information, Figure S1). During the experiments, the temperature was kept constant with a thermostatic water bath, Grant Instrument, GD-120. Before every experiment, the samples were outgassed at 300 °C for 2.5 h. The regeneration of the materials was carried out in the following experimental conditions: in two consecutive adsorption cycles, the samples were outgassed under vacuum at room temperature or under vacuum at 150 °C for 1 h.

## 4. Conclusions

The present study showed that it is possible to prepare activated carbons from glycerin and dopamine that present high adsorbed amounts and high selectivity values for separation that involve gas molecules such as ethane, ethylene, carbon dioxide, and me-thane. The mass ratio between the activating agent (KOH) and the carbonized material was critical for the adsorbent properties. In fact, mass ratios KOH:Carbonized higher than one are often used to promote the highest values of surface area and microporous volumes. Nevertheless, an extremely developed microporosity, at the cost of very narrow pores, is not necessarily the best option, as demonstrated by the present work. Here, we studied materials with mass ratios of KOH:Carbonized of one and less and concluded that the best option was a ratio of 0.75, since high adsorbed amounts and high selectivity values were achieved with this material for the systems ethane/ethylene, carbon dioxide/methane, and ethane/methane. Moreover, lower amounts of activating agent also correspond to a more economical and environmentally friendly preparation procedure.

## Figures and Tables

**Table 1:** Elemental analysis (CHNS analysis) in % for glycerin and for the activated carbons. Oxygen content was obtained by the difference between the total percentage (100%) and the sum of percentages (wt %) of carbon, hydrogen, sulfur, and nitrogen. The pHpzc values for the activated carbons are also indicated.

Sample →	Glycerin	Gdop1.0	Gdop0.75	Gdop0.50	Gdop0.25
C (%)	39.1	60.1	62.1	59.1	65.1
H (%)	8.7	2.5	2.0	2.1	2.1
N (%)	-	1.1	0.9	0.8	0.8
S (%)	-	6.1	7.2	12.1	13.1
O (%)	57.5	30.2	27.8	12.1	13.1
pH_PZC_	-	1.9	2.0	2.4	3.2

**Table 2:** Textural characteristics of glycerin–dopamine carbons evaluated by N_2_ adsorption isotherms, apparent-tap-densities (in g·cm^−3^) and yield of the preparation.

Samples	→	GdopC	Gdop1.0	Gdop0.75	Gdop0.50	Gdop0.25
Partial yield (%)	Carbonization	49	-	-	-	-
	Activation	-	42	52	56	64
Global yield (%)		-	3.4	5.3	5.7	5.9
Apparent-tap-density		0.65	0.12	0.42	0.52	0.52
*A*_BET_ (m^2^ g^−1^)		<10	1657	1276	834	227
V_TOTAL_ (cm^3^ g^−1^)		-	0.73	0.56	0.37	0.12
V_MESO_ (cm^3^ g^−1^)		-	0.10	0.06	0.05	0.03
V_MICRO_ (cm^3^ g^−1^)		-	0.63	0.48	0.32	0.09

## Data Availability

The data presented in this study are available on request from the corresponding author.

## Supplementary Materials

The following supporting information can be downloaded at: https://www.mdpi.com/article/10.3390/molecules28104071/s1, Section S1: Adsorption apparatus for the adsorption of ethane, ethylene, carbon dioxide, and methane at pressures up to 5 bar; Section S2: FTIR and SEM for glycerin–dopamine carbonized; Section S3: Selectivity values and X-Y diagrams in the Gdop0.75 for the separation of ethylene/carbon dioxide; Section S4: Selectivity values and X-Y diagrams in the Gdop0.75 for the separation of ethylene/carbon dioxide; Section S5: SEM for the sample Gdop0.75.
