# Supplementary material for: Novel Carbonaceous Adsorbents Prepared from Glycerin Waste and Dopamine for Gas Separation
Source: Molecules. 2023 May 13;28(10):4071. doi: 10.3390/molecules28104071 (PMC10221774; doi:10.3390/molecules28104071)
Supplement: Supplementary file 1 [file molecules-28-04071-s001.zip › molecules-2367603-supplementary.pdf]

Supporting Information

Novel carbonaceous adsorbents prepared from glycerin waste and dopamine for gas separation

Mary Batista[1], Renato Carvalho[2], Moisés L. Pinto[3], João Pires[1,*]

[1]CQE, Centro de Química Estrutural, Institute of Molecular Sciences, Departamento de Química e Bioquímica, Faculdade de Ciências, Universidade de Lisboa, Campo Grande, 1749-016 Lisboa, Portugal

[2]IBEROL, Sociedade Ibérica de Biocombustíveis e Oleaginosas, S.A., 2600-531 Alhandra, Portugal

[3]CERENA, Departamento de Engenharia Química, Instituto Superior Técnico, Universidade de Lisboa, 1049-001 Lisboa, Portugal

* *Correspondence*: jpsilva@ciencias.ulisboa.pt

Section S1. Synthesis of the glycerin-dopamine carbon materials.

Section S2. Adsorption apparatus for the adsorption of ethane, ethylene, carbon dioxide and methane at pressures up to 5 bar.

Section S3. FTIR and SEM for glycerol-dopamine carbonized.

Section S4. Selectivity values and X-Y diagrams in the Gdop0.75 for the separation of ethylene/carbon dioxide.

Section S5. SEM for the sample Gdop0.75

Section S1. Synthesis of the glycerin-dopamine carbon materials.

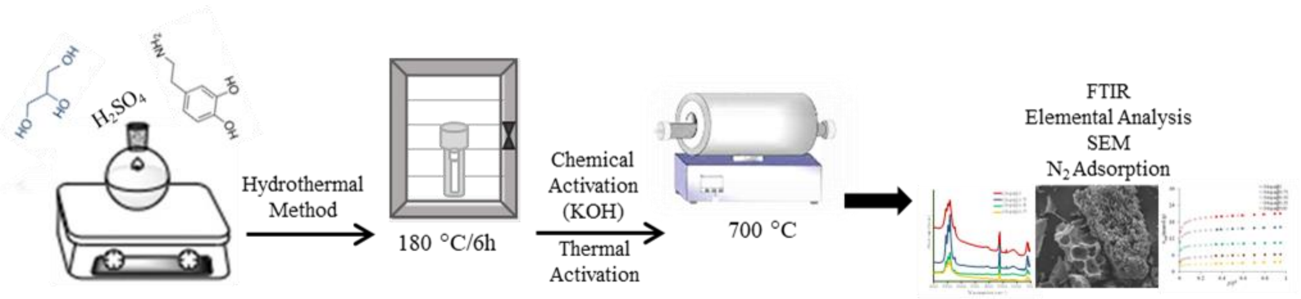

Figure S1. Schematic illustration of the preparation of glycerin-dopamine carbon materials.

Section S2. Adsorption apparatus for the adsorption of ethane, ethylene, carbon dioxide and methane at pressures up to 5 bar.

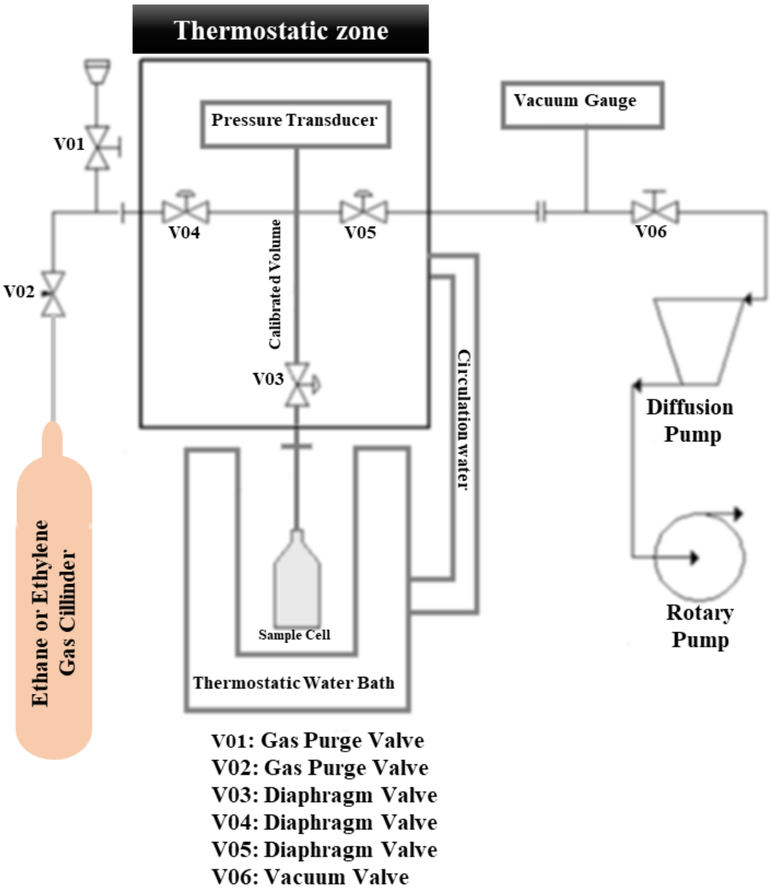

Figure S2. Gas adsorption system (used for the determination of adsorption isotherms at high-pressure).

Section S3. FTIR and SEM for glycerin-dopamine *carbonized*.

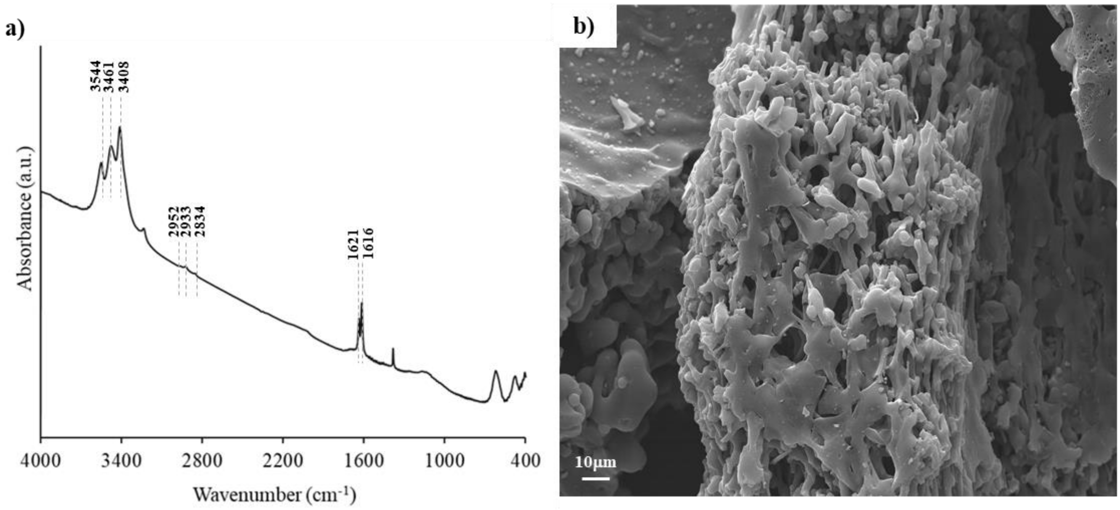

Figure S3. (a) FTIR spectra and (b) SEM image of the glycerol-dopamine *carbonized* (GdopC).

Section S4. Selectivity values and X-Y diagrams in the Gdop0.75 for the separation of ethylene/carbon dioxide.

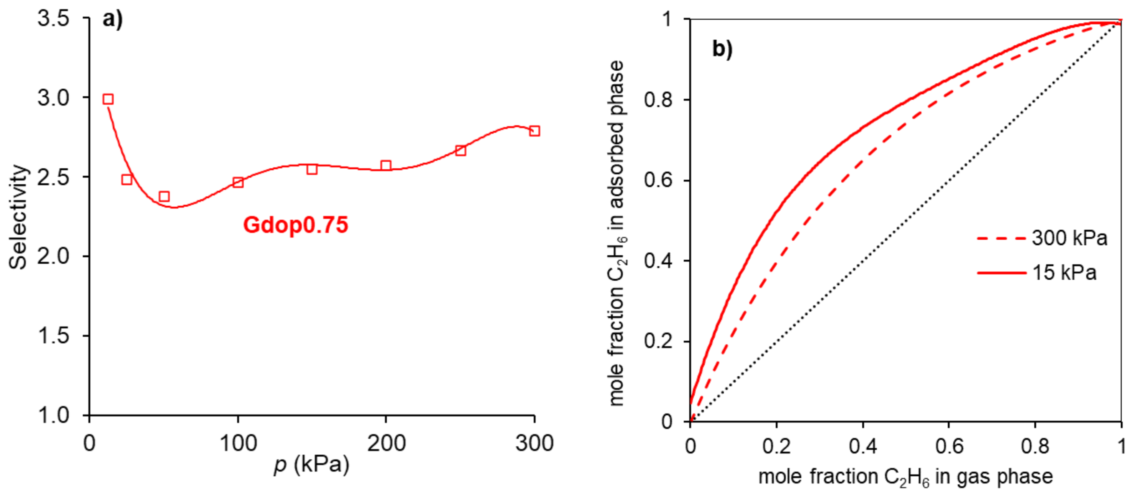

Figure S4. (a) Selectivity values and (b) X-Y diagrams in the Gdop0.75 for the separation of ethylene/carbon dioxide, estimated by the Ideal Adsorbed Solution Theory (IAST).

Section S5. SEM for the sample Gdop0.75

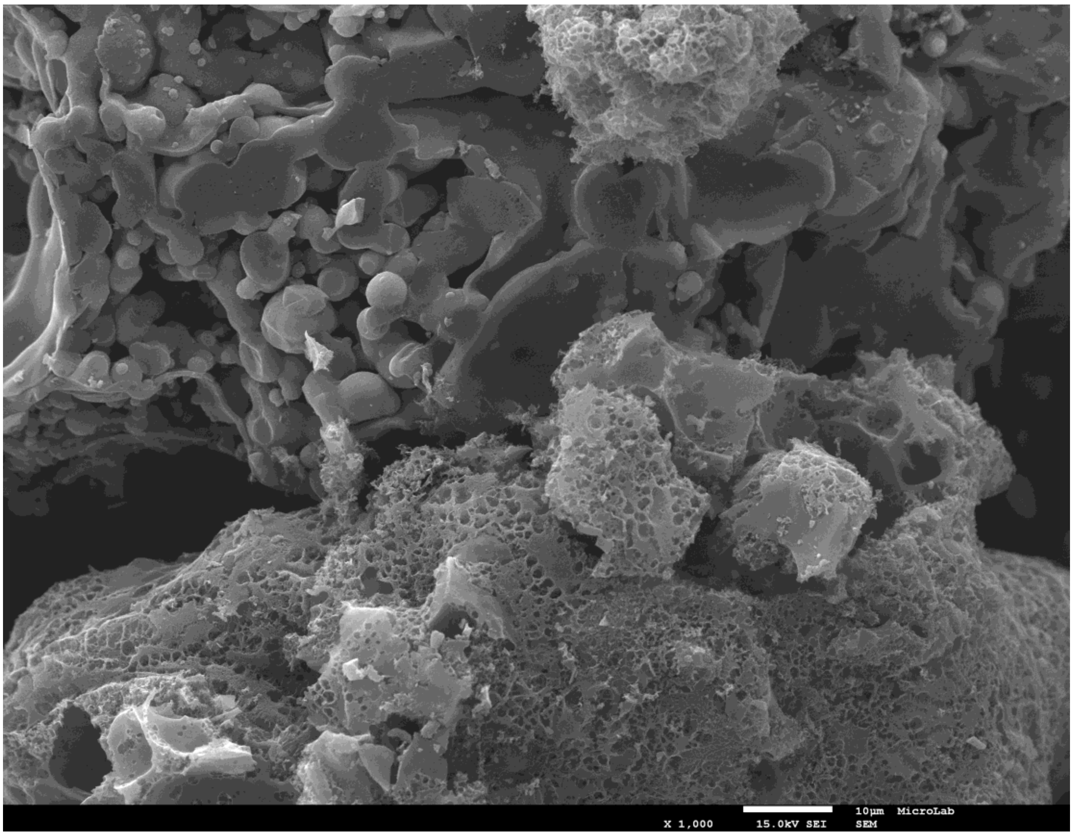

Figure S5. SEM for the sample Gdop0.75.
